# Expectations and Motivations for Participation in Clinical Trials Utilizing Psychedelics for Treatment‐Resistant Depression: A Qualitative Study

**DOI:** 10.1002/brb3.71544

**Published:** 2026-06-16

**Authors:** Christopher Poppe, Liisa Lyck, Laura Bechtold, Dimitris Repantis

**Affiliations:** ^1^ Department of Psychiatry and Neurosciences Campus Charité Mitte Charité–Universitätsmedizin Berlin Berlin Germany; ^2^ German Center for Mental Health (DZPG), Partner Site Berlin‐Potsdam Germany

**Keywords:** clinical trials, expectations, motivation, psychedelic treatments

## Abstract

**Purpose:**

Expectations strongly shape participant experiences in clinical trials, contributing to placebo effects that complicate interpretation of outcomes. While quantitative tools exist to measure expectations, their subjective and multidimensional nature, especially for individuals with treatment‐resistant depression (TRD), remains underexplored. This study aimed to explore the motivations and expectations of participants in psychedelic clinical trials.

**Method:**

Seventeen semi‐structured interviews were conducted with individuals prior to screening for two TRD clinical trials involving 5‐MeO‐DMT or psilocybin at a German university hospital. Two matched follow‐up interviews were also completed after trial participation. Data were analyzed thematically to identify overarching themes and subthemes.

**Findings:**

Two main themes emerged: motivations and expectations, each with four subthemes. Motivations included hope, demoralization, prior psychedelic experience, and social motivation. Expectations involved anticipated symptom reduction, perceived mechanisms of change during the psychedelic experience, the role of setting, and retrospective expectations. Many participants viewed trial participation as a last resort due to chronic illness and previous treatment failures. In two cases, retrospective accounts suggested that initially cautious expectations were reinterpreted as stronger after participation.

**Conclusion:**

Expectations in psychedelic trials are complex and may evolve over time. These findings highlight the need to systematically address expectancy in trial design, informed consent, and interpretation of clinical outcomes, particularly in treatment‐resistant populations.

## Graphical abstract

Individuals with treatment‐resistant depression considering psychedelic trial participation were motivated by chronic illness, failed treatments, and hope for a novel intervention, while actively minimizing expectations to guard against disappointment. Post‐trial interviews revealed that stronger hopes had been present all along, highlighting the complex, evolving nature of expectancy and the need for tailored assessment and informed consent practices.

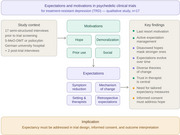

## 1 Introduction

In psychedelic research, pre‐trial expectations are often described as part of the “set” (Hartogsohn 2016) and can significantly influence both the subjective experience and treatment outcomes. Generally, expectancy is a key part of the placebo effect and is thought to shape outcomes in psychedelic trials, aligning with the idea of psychedelics as “active super‐placebos” (Dupuis and Veissière 2022). This is aggravated by the fact that for the evaluation of treatment outcomes, double‐blind controlled trials with psychedelic substances (henceforth, psychedelic trials) are difficult due to the substances’ unmistakable subjective effects, which most often lead to unblinding. As a result, placebo and nocebo effects are common and can complicate interpretation of trial results (Muthukumaraswamy et al. 2021).

Expectations are, however, not merely an influence on trial outcomes but also a potential mechanism of therapeutic change itself. They are considered a core “common factor” in psychotherapy research (Wampold 2015), which has led to clinical strategies like using informed consent to shape patient expectations (Ladwig et al. 2024). The link between a patient's baseline expectancy and their eventual improvement in treatment is debated, and a recent study demonstrates that the trajectory of expectancy is also critical (Rozenkrantz et al. 2025). This is of particular importance for psychedelic therapy because “expectations may be reset under the action of psychedelic drugs and MDMA treatments” (Colloca et al. 2023). Importantly, the degree to which a patient's expectations change during psychotherapy was found to be an independent predictor of recovery from depression (Rozenkrantz et al. 2025).

In general, several tools exist to measure expectancy, such as the Credibility/Expectancy Questionnaire (CEQ; Devilly and Borkovec 2000), which distinguishes between cognitive and affective components, and the Stanford Expectations of Treatment Scale (SETS; Younger et al. 2012). However, while these are quick and psychometrically sound, they are also underutilized in psychedelic trials. Furthermore, these standardized measures, while helpful, may also not fully capture the complexity of expectancy in psychedelic trials. Muthukumaraswamy et al. (2021) and Noorani et al. (2023) argue for a purpose‐built scale, suggesting expectancy should be treated as a multidimensional construct. They propose key distinctions, including a differentiation between expectation of subjective effects and treatment outcome, between the mechanisms of change and the “meaning response,” as well as a difference between hope and expectation (Noorani et al. 2023). According to Noorani et al. (2023), a tailored approach would better reflect the unique dynamics of psychedelic therapy and improve the assessment of expectations in treatment outcomes.

In studies of healthy volunteers, Ley et al. (2025) employed qualitative content analysis across multiple information sources, including brief open‐ended questions and questionnaire data, to examine motivations for participation in psychedelic trials. They found that participants were motivated, among other factors, by interests in personal development and transformational experiences. Donegan et al. (2025) similarly combined qualitative content analysis with the CEQ to investigate expectations among participants with depression in an LSD microdosing trial. Using pre‐ and post‐trial interviews with 12 participants in a psilocybin‐assisted psychotherapy trial for methamphetamine use disorder, Brett and Colleagues (2025) applied interpretative phenomenological analysis to explore the experiential arc of trial participation, reporting that participants generally held tempered expectations regarding therapeutic benefit.

Despite being a major population in psychedelic clinical trials, individuals with treatment‐resistant depression (TRD) remain underrepresented in qualitative research on expectations and motivations. A notable exception is the study by Breeksema et al. (2024), which retrospectively highlighted challenges in expectation management of trial participants. Prospective studies, without the change of expectations through trial participation, are still limited. This gap is notable given that the chronicity of TRD and repeated treatment failures likely shape expectations in distinctive ways, for example, through severe desperation alongside hope for novel interventions (Basedow et al. 2025). Qualitative methods are particularly suited for capturing the lived experience of psychiatric illness and the subjective complexity of expectations, making them well‐positioned to address this gap. The present study therefore aims to explore the motivations and expectations of individuals with TRD considering participation in two psychedelic clinical trials, both prospectively and retrospectively.

## 2 Methods

The qualitative methods described in the next sections adhere to the consolidated criteria for reporting qualitative studies (COREQ‐32) checklist that can be found in the Supporting Information Material.

### 2.1 Recruitment

We recruited by purposive sampling from potential participants for two psychedelic trials for TRD. In the first clinical trial participants received a single dose of low, medium, or high‐dose 5‐MeO‐DMT together with psychological support which was provided throughout the trial. The second trial investigated low, medium, and high doses of psilocybin for TRD also with psychological support provided throughout the trial. Importantly, both trials entailed an open‐label extension making it possible to receive the psychedelic substance independent of randomization. Participants first had a telephone call with members of the clinical study team and were asked whether they would be willing to also participate in this interview study. If they agreed, they received study information, which was again discussed during informed consent. While we recruited for this study from potential participants for psychedelic trials, interested participants were not necessarily included in the trials. The recruitment for the pre‐trial interviews took place from May 2024 to May 2025 and resulted in 17 interviews. From the 17 pre‐trial participants, only three participated in a psychedelic trial and two were open to another post‐trial interview.

### 2.2 Sampling

The sample size of 17 participants was considered appropriate for this explorative and descriptive study using thematic analysis. In line with qualitative principles, sample adequacy was determined by information power rather than numerical thresholds (Malterud et al. 2016). Given the focused research question, the specificity of the sample, and the specific interview structure, we consider the sample to provide sufficient information to preliminarily map motivations and expectations in this population, while acknowledging that the short interview duration limits the depth of information power that can be claimed.

### 2.3 Study Design

Prior to screening for two psychedelic trials, we conducted semi‐structured interviews using an interview guide that included a range of questions and prompts addressing participants’ expectations of trial participation (see Table 1). In addition, two participants were re‐interviewed after completing both the pre‐trial interview and the trial itself (for the interview guide, see Table 2). For these cases, we conducted a retrospective within‐case analysis to examine participants’ initial expectations and how this evolved following trial participation.

**TABLE 1:** Semi‐structured interview guide pre‐trial.

1. Please tell me how you came to participate in the study. 2. Can you describe the course of your depressive disorder? 3. What prior treatments have you received, and what effect (beneficial/unbeneficial) did they have? 4. How did you learn about the study? 5. Why did you decide to participate in the study? 6. What do you expect from… (the dosing session, transformation during the study, after the end of the study)? 7. What do you hope to gain from participating in the study? 8. What do your friends or family think about your potential participation in the study? 9. Have you ever tried psychedelic substances? (Prompts: experiences, importance of experiences for trial participation, knowledge and source of knowledge) 10. Is there anything else relevant that hasn't been mentioned yet?

**TABLE 2:** Semi‐structured interview guide post‐trial.

**1**. How did you come to participate in the study? **2**. How did you find out about the study? **3**. Why did you decide to participate in the study? **4**. What did you hope to gain from participating in the study? 5. Can you describe what happened during the dosing session(s)? **6**. What did you feel? Prompt: Internal changes, feelings, emotions, memories **7**. What significance do these experiences have for you? **8**. What was the experience with the therapists like? Prompt: safety, treatment room, preparation, negative and positive. **9**. What was the therapists' presence like during the dosing session? **10**. How did you feel after the dosing session? **11**. What was the time like after the study ended? (Effect on depression, etc.) **12**. What are the most significant changes compared to before the start of this study? **13**. If you had the opportunity to change something about the treatment, what would you do differently? **14**. Has the study changed you as a person or your worldview? **15**. What advice would you give someone considering participating in a study or therapy with psychedelics? **16**. What would this person need to know to be ready to participate? **17**. What would you have liked to know beforehand? **18**. What was it like talking to other people about the study? **19**. Is there anything else you would like to add?

For the minimization of participant burden, brief 15‐min interviews were planned before the trial. After the trial, interviews were not limited in length. Interviews were audio recorded and transcribed verbatim. We first used noScribe (Dröge 2024) to automate transcription. Transcripts were corrected by two research assistants, after which the first author read and checked each interview in‐depth to verify the content.

### 2.4 Data Collection

The pre‐trial interviews were conducted in a therapy room in a hospital setting and lasted a mean of 13 min (range: 7–20 min). Two post‐trial interviews were conducted with participants who had also participated pre‐trial. The post‐trial interviews lasted a mean of 37.5 min (range: 32–43 min).

In two cases in the pre‐trial interviews, a screening clinician (D.R.) was present during the interview so that participants would not have to give information redundantly. The aim of these interviews was to explore the participants’ expectations and motivations, and how they made sense of potentially participating in a psychedelic trial, as well as their trial experience after they had participated (see Tables 1 and 2). Due to the explorative nature of the study, the brevity of the interviews was deemed adequate (see Section 4.6 also).

The interviews were conducted by C.P., a male clinical psychologist with a PhD; D.R., a male psychiatrist with an MD; and L.B., a female psychotherapist with an MSc. None of them had prior relationships to the participants, and apart from the study information, there was no information provided about the interviewers.

The shortness of the pre‐trial interviews was due to pragmatic and ethical considerations of minimizing participant burden prior to confirming eligibility for the psychedelic trial, so as not to require extensive time investment before participation was assured.

### 2.5 Data Analysis

As our study was exploratory in nature, we used a descriptive design to analyze the themes reported by the participants. We used reflexive thematic analysis following the steps outlined by Braun and Clarke (2006). Following in‐depth reading of the transcripts, one author (C.P.) coded every sentence or paragraph in the interview inductively in MAXQDA (VERBI 2025). This resulted in 137 inductive codes.

Each code was independently reviewed by a second author (L.L.), and a reflexive discussion led to resolution of disagreements. Then, both C.P. and L.L. grouped inductive codes to form 39 higher‐level codes. These codes then formed a codebook, in which codes were ascribed to categories and refined through discussion between the two authors (C.P. and L.L.). These categories were ascribed to the two themes describing expectations and motivations. Categories of codes were identified across the dataset and became recurrent, with no substantially new codes and categories emerging in later interviews, indicating sufficient depth and analytic adequacy for the study aims. In Supporting Information Material 1, we provide a codebook with definitions.

### 2.6 Reflexivity

The research team consisted of clinicians and researchers with backgrounds in medicine and psychiatry, psychotherapy, psychedelic research, and medical ethics, embedded within a psychiatric university hospital. In two cases a screening clinician (D.R.) was present during the interview, and in three cases a screening clinician (L.B. or D.R.) conducted the research interview, creating potential role conflicts and power asymmetries that may have influenced data generation, including through social desirability in participant responses. The overlap between clinical and research roles was most pronounced in cases where the screening clinician also conducted the interview, as participants may not have felt fully able to separate the two contexts. Generally, our pre‐existing clinical experience and knowledge of psychedelic‐assisted therapies, including assumptions about therapeutic roles and anticipated participant expectations, may have shaped both the formulation of interview questions and the interpretation of the data. To address this, interviews followed a semi‐structured guide to support consistency across interviewers, and participants were explicitly informed that interview responses were confidential and would not affect trial eligibility or clinical decision‐making. Throughout the analytic process, the coders engaged in ongoing reflexive discussions to critically examine how clinical perspectives and expectations may have shaped coding and theme development. Themes were iteratively reviewed and refined through dialogue.

### 2.7 Ethical Considerations

The study was approved by the ethics commission of Charité (EA4/008/24), and all procedures adhered to the Declaration of Helsinki. All participants provided written informed consent, which outlined the voluntary and uncompensated nature of their participation, their right to withdraw at any time, and guarantees of confidentiality. To protect participant anonymity, transcripts and identifying documents were stored separately.

## 3 Results

For the 17 interviews, the comprehensive characteristics of the sample can be found in Table 3. The resulting themes from the thematic analysis are ordered into two main themes: *Motivations for Psychedelic Trial Participation* and *Expectations for Psychedelic Trials*. For the codebook, including participant quotes for each code, see Supporting Information Material 2.

**TABLE 3:** Sociodemographic characteristics of participants.

Characteristics	Pre‐trial	Post‐trial	
	*n*	*n*	
Gender			
Female	9	2	
Male	8	0	
Duration of illness			
Less than 5 years	3	0	
Five to 10 years	2	0	
More than 10 years	12	2	
Numbers of lifetime antidepressant medications			
2 Medications	5	0	
3–4 Medications	8	2	
5–6 Medications	4	0	
Somatic comorbidites (e.g., chronic pain, diabetes, hashimoto)	7	1	
Previous psychedelic experience	7	2	
Ketamine/ECT/TMS and other experimental treatments	1	0	

*Note*: *N*  = 17. Participants were on average 37 years old (SD  = 9.83).

In the following, the themes are described textually.

### 3.1 Motivations for Psychedelic Trial Participation

This theme describes motivations of participants with TRD who showed interest in participating in a psychedelic trial. It encompasses the subthemes of *demoralization*, *hope*, *previous psychedelic experiences*, and *social motivation*.

#### 3.1.1 Hope

Participants obtained hope through former experiences with psychotherapy and psychedelic substances (Quotes 1 and 2). The return of hope in general was a motivation for trial participation (Quote 3). Participants held positive views regarding the substance because they perceived it as natural, even though the trial utilized synthetic compounds. The medical setting contributed to this optimism by providing a sense of safety (Quote 4). Furthermore, participants compared their expectations for psychedelic therapy to the known effects of antidepressant medications (Quote 5). Specifically, participants sought relief from the emotional blunting associated with SSRIs and expressed a desire to experience joy in the context of anhedonia. Participation in the clinical trial was motivated by the perception of the psychedelic intervention as a treatment of last resort (Quote 6).

#### 3.1.2 Demoralization

The subtheme of Demoralization describes the chronic suffering caused by the depressive illness and the experience of unsuccessful treatments. Furthermore, consistent with the definition of treatment resistance, previous antidepressant treatments had failed to ameliorate symptoms. Participants reported trying multiple medications without subjective improvement. Moreover, participants noted that side effects and discontinuation symptoms exacerbated their suffering. (Quote 7). Participants reported a range of impairing depressive symptoms, including anhedonia, hopelessness, and social withdrawal. Several participants noted that their sense of hopelessness increased as the duration of the illness persisted (Quotes 8 and 9).

#### 3.1.3 Previous Psychedelic Experiences

Previous psychedelic experiences acted as a motivating factor for trial participation. Participants reported recreational use and use with therapeutic intent for the purpose of experiencing a novel experience (Quote 10) or “ego death” (ego dissolution) (Quote 11). Some participants described independent experimentation, such as microdosing. Others reported tapering off antidepressant medication to safely self‐administer psychedelics in a manner similar to trial conditions. Conversely, participants who had previously taken part in psilocybin trials expressed disappointment regarding the therapeutic results and the lack of post‐trial care (Quote 13). Participants also viewed previous substance use, including cannabis, as a model for the psychedelic experience. These prior experiences motivated participants for the psychedelic trial participation.

#### 3.1.4 Social Motivation

Support from friends and family influenced the motivation to participate in the trial. Participants reported that encouragement from their social network, including parents, supported their decision (Quote 15). However, views within social networks were not always uniform. Some participants reported that friends held differentiated views regarding benefits of specific psychedelic substances (Quote 14). Others experienced stigmatization and skepticism from friends and family members (Quote 16).

### 3.2 Expectations for Psychedelic Trials

The thematic analysis resulted in four subthemes of expectations: *Symptom Reduction*, *Mechanism of Change in the Psychedelic Experience*, *Setting*, and *Expectations in retrospect*.

#### 3.2.1 Symptom Reduction

The theme of *Symptom Reduction* encompasses the primary motivation for participants entering the psychedelic trial: the hope for relief from persistent and often debilitating symptoms (Quote 17; see theme Demoralization above). However, shaped by a history of ineffective prior treatments, participants did not anticipate that symptom reduction would be straightforward and expected only “small changes” (Quote 20). Their expectations were rarely for a simple, neurobiologically mediated decrease in depressive symptoms, but rather for a “fundamental” shift in their condition (Quote 20) or a “breakthrough” (Quote 22). However, some participants expected a “cure” that would immediately fix their depression (Quote 19). Instead, they engaged in active expectation management, often minimizing their hopes to guard against potential disappointment (Quote 18). This protective strategy, rather than indicating indifference, underscores the profound weight they placed on the trial's potential success, as is also evident in the theme of *Expectations before and after* (see below).

#### 3.2.2 Mechanism of Change in the Psychedelic Experience

Participants reported diverse expectations regarding how the psychedelic experience might lead to change. Some expected to feel different under the effects of 5‐MeO‐DMT, for example, to feel “ego death” (Quote 23), feeling closer to themselves or the world (Quotes 24 and 25), or to feel trust (Quote 26), which then would reduce symptoms. Others stated that they had no clear idea of how the psychedelic experience could be beneficial. Several participants articulated specific assumptions about mechanisms of action. These included neurobiological explanations, such as increased brain connectivity or changes in neural functioning (Quote 31). Some participants held detailed phenomenological and neuroscientific preconceptions, including expectations of ego dissolution (Quote 23) and its relation to the default mode network. In contrast, other participants emphasized psychological mechanisms and expressed more general hopes for an experience that would provide material they could work with therapeutically, rather than a specific experiential outcome (Quotes 29 and 30). This included expectations that the psychedelic experience might facilitate the emergence or recovery of emotionally salient or traumatic memories (Quotes 27 and 28).

#### 3.2.3 Therapists and Setting

Participants expressed strong expectations for a professional, safe, and supportive setting (Quotes 33, 37, 38), with therapists perceived as a central component of this environment. Expectations toward therapists were often framed in relation to the limits of clinical trials and to participants’ previous therapeutic relationships (Quote 32). Some anticipated experiencing anxiety during the psychedelic session and expected that trust in the therapist would help mitigate both this anxiety and uncertainty toward the experimental treatment (Quote 34). The therapeutic setting was frequently expected to be a holding environment that distinguished the clinical context from taking psychedelic substances alone (Quote 36). One participant with prior personal use of psychedelics expressed the view that therapists’ own psychedelic experience could be valuable, arguing that the ineffability of psychedelic experiences requires experiential understanding (Quote 35). However, this preference for therapists with personal psychedelic experience was voiced by only one participant.

#### 3.2.4 Expectations Before and After

Interviews conducted with two participants after trial participation provided additional insight into expectation management over time. In the pre‐trial interview, one participant emphasized that they did not expect to be cured and actively disavowed having strong expectations (Quote 39). In the post‐trial interview, the same participant reflected on this stance and described a reassessment of their earlier expectations (Quote 40). They reasoned that, despite consciously framing their expectations as modest or absent prior to participation, stronger hopes for benefit had nevertheless been present (Quote 40). The second participant similarly highlighted the changing nature of expectations. Before the trial, this participant described primarily expecting to have an experience, whereas in retrospect they emphasized a desire for even small improvements and the importance of having tried all available treatment options (Quotes 41 and 42). Together, these two cases illustratively suggest how expectations may be retrospectively reinterpreted following trial participation and how pre‐trial expressions of limited expectations may mask more substantial hopes that become more apparent in hindsight.

## 4 Discussion

This qualitative study investigated the motivations and expectations of individuals with TRD who were considering participation in a psychedelic therapy trial. The results can be understood against the backdrop of demoralization and re‐moralization and have implications for further research.

### 4.1 Demoralization and Different Mechanisms of Change

The findings reveal that participants' primary motivations stemmed from the chronicity of their condition (“Demoralization”) and a history of treatment failures, which fits into the wider literature on demoralization (Clarke and Kissane 2002). In this light, psychedelic trials offer participants hope for therapeutic change through experimental treatment. However, participants’ expectations were nuanced and extended beyond a simplistic notion of a “magic pill,” rather expecting a process of change. Our findings also show participants' different subjective folk theories regarding the mechanism of this change. Participants articulated a range of anticipated mechanisms, spanning neurobiological theories (e.g., enhanced brain connectivity), psychological processes (e.g., ego dissolution and trauma processing), and existential shifts (e.g., increased connectedness). Recent findings in research on expectations in microdosing research also underlined that participants in clinical trials share neurobiological theories (cf. “neural rewiring;” Donegan et al. 2025).

This heterogeneity in subjective theory of change necessitates a flexible, patient‐centered therapeutic stance. Such a collaborative approach is central to established models of psychedelic‐assisted therapy (Brennan and Belser 2022) and involves exploring a patient's expectations within the preparatory sessions rather than imposing a single theoretical model which counteracts “poor‐fitting interpretations that may disrupt positive treatment outcomes” (Brennan and Belser 2022).

### 4.2 The Therapist(s) as the Setting

Furthermore, our findings underscore the centrality of “set and setting” (Hartogsohn 2017). The qualitative data suggest that for this cohort, the ‘setting’ is construed both as a medicalized safety net in a reputable university clinic and as a relational construct with trust in the therapists. This interpersonal trust was perceived as a prerequisite for therapeutic engagement, functioning as a necessary condition for participants to navigate potentially challenging experiences. The rare preference for therapists with personal psychedelic experience highlights a key consideration in therapist training, suggesting that patients may value a therapist's perceived capacity for understanding of altered states of consciousness. However, personal psychedelic experiences by therapists present ethical challenges (Villiger 2024; Poppe et al. 2025).

### 4.3 Expectations, Psychedelic Experience, and Outcome: An Unclear Relationship

Situating these findings within the broader context of quantitative psychedelic research, a recent review by Weiss et al. (2024) highlighted a significant gap in the literature, noting that only two psychedelic studies have utilized an expectancy measure, with both using single‐item, non‐standardized tools. One of these studies by Szigeti et al. (2024) measured expectancy in a trial comparing escitalopram and psilocybin for depression. Before dosing, patients rated their expected symptomatic improvements for each treatment. The results indicated that patients held significantly higher expectations for psilocybin therapy than for escitalopram. However, a significant correlation between pre‐treatment expectations and therapeutic response was found only in the escitalopram group, not in the psilocybin group. Nonetheless, expectancy appears influential in personality change following psychedelic use outside of clinical settings. For example, a study on naturalistic settings with ayahuasca users by Weiss et al. (2021) found that individuals anticipated favorable personality changes in domains such as neuroticism and extraversion.

A key finding from the current study is that participants actively managed their expectations, but also that they might disavow expectations before the trial (as present in the pre‐ and post‐trial interviews). They frequently described minimizing their expectations, a psychological strategy likely employed to mitigate potential disappointment arising from a long history of unsuccessful treatments. This observation is consistent with recent findings from Breeksema et al. (2024), who identified a similar theme of expectation management. Their research showed also that some participants held highly specific expectations, such as gaining certain insights or connecting to emotions, and expressed disappointment when the experience did not meet these expectations.

### 4.4 Informed Consent and Therapeutic Misconception

Despite personal expectation management by participants, the potential for heightened and unrealistic expectations, often fueled by enthusiastic media narratives (Yaden et al. 2022), necessitates thorough disclosure in the informed consent process to ensure that therapeutic misconception is not the case and clinical equipoise is upheld by researchers. Consequently, the informed consent process should not only address and temper potentially high expectations but also remain sensitive to the hope that might exist despite the participants’ overt statements (“Disavowed expectations”).

In the context of TRD, this is even more the case because of demoralization. As Basedow et al. (2025) have shown, “higher depression severity was linked to more favorable expectations of ketamine‐ and psilocybin‐assisted therapy.” In agreement with the authors, this qualitative study underlines the need for thorough informed consent practices that address participant expectations.

### 4.5 Future Research

Future research should aim to systematically investigate whether specific types of expectations, or the congruence between expectation and experience, correlate with clinical outcomes, thereby informing the tailoring of therapeutic preparation. This investigation could potentially extend beyond the advice by the FDA in their Complete Response Letter for their rejection of MDMA‐assisted therapy by MAPS/Lykos Therapeutics, which required an “assessment of participant expectancy at baseline” (US FDA 2025) to minimize bias.

Finally, the complexity of participant expectations in this study has significant methodological implications. Standard expectancy scales may lack the specificity to capture the unique phenomenological and therapeutic domains relevant to psychedelic‐assisted therapy, such as anticipated mystical‐type experiences, psychological insight, or emotional catharsis. Consequently, these findings suggest a rationale for the development and validation of a psychometric instrument designed to measure expectancy in psychedelic trial populations, as already argued by Muthukumaraswamy et al. (2021) and Donegan et al. (2025). This measure could potentially enable the quantification of expectations among both participants and investigators, facilitate comparisons between psychedelic‐naïve and psychedelic‐experienced cohorts, and allow for empirical testing of the moderating or mediating role of expectancy on clinical outcomes. The present findings tentatively suggest several dimensions such a measure might capture: the disavowed expectations finding points to the need for items assessing implicit or minimized hopes alongside explicitly stated ones, while the diversity of anticipated mechanisms of change suggests distinguishing between neurobiological and psychological expectation types as separate constructs.

### 4.6 Limitations

Our study is limited in scope. The short interview duration (mean 13 min) was deemed adequate for the explorative research question but may have limited the depth of exploration of expectations pre‐trial, and findings should be interpreted accordingly. The sample was recruited from individuals interested in psychedelic trials at a single university hospital in Germany, which limits generalizability to other clinical and cultural contexts. Furthermore, individuals with a more optimistic attitude toward psychedelic interventions may have self‐selected for both trial and interview participation, which may contribute to the relative homogeneity of findings regarding anticipated outcomes. This self‐selection is inherent to the population of interest, however, as willingness to consider psychedelic trial participation is itself a defining characteristic of this group.

In two pre‐trial interviews, the screening clinician was present and may have introduced social desirability bias, potentially constraining participants' expression of ambivalence or skepticism. This concern was amplified in the case where the screening clinician also conducted the interview, as participants may not have felt fully able to separate the interview context from the eligibility assessment.

The longitudinal component of the study is extremely limited, with only two participants completing post‐trial interviews. Findings from these cases should therefore be understood as illustrative rather than representative of how expectations evolve across trial participation. We also have no data on how pre‐trial expectations influenced trial outcomes, which remains an important question for future research. Finally, the sample included both psychedelic‐naive and psychedelic‐experienced individuals, and while this provides nuance, future research could compare these groups more systematically.

## Author Contributions

**Christopher Poppe**: Conceptualization, methodology, software, data curation, formal analysis, validation, visualization, writing – review and editing, writing – original draft, investigation. **Liisa Lyck**: Conceptualization, investigation, writing – review and editing, methodology, validation, data curation, software, formal analysis, writing – original draft. **Laura Bechtold**: Conceptualization, investigation, writing – review and editing, methodology, formal analysis, supervision, project administration. **Dimitris Repantis**: funding acquisition, investigation, writing – review and editing, validation, methodology, formal analysis, project administration, supervision, resources.

## Funding

The authors have nothing to report.

## Ethics Statement

The study was approved by the ethics commission of Charité Berlin (EA4/008/24), and all procedures adhered to the Declaration of Helsinki. All participants provided written informed consent, which outlined the voluntary and uncompensated nature of their participation, their right to withdraw at any time, and guarantees of confidentiality. To protect participant anonymity, transcripts and identifying documents were stored separately.

## Conflicts of Interest

The institution of DR has received research support from MAPS Europe B.V., COMPASS Pathways plc., Beckley Psytech ltd, Definium Therapeutics Inc., and Helus Pharma Inc. DR and LB are founding members and honorary Board Members of MAPS Deutschland, a Germany‐based non‐profit research and educational organization associated with MAPS. The other authors declare no conflicts of interest.

## Supporting information




**Supplementary Material**: brb3715440‐sup‐0001‐SuppMat.docx

**Supplementary Material**: brb3715440‐sup‐0002‐SuppMat.docx

## Data Availability

Anonymized data are available from the authors at reasonable request.
