# Supplementary material for: Expectations and Motivations for Participation in Clinical Trials Utilizing Psychedelics for Treatment‐Resistant Depression: A Qualitative Study
Source: Brain Behav. 2026 Jun 16;16(6):e71544. doi: 10.1002/brb3.71544 (PMC13272625; doi:10.1002/brb3.71544)
Supplement: Supplementary file 1 — Supplementary Material: brb3715440‐sup‐0001‐SuppMat.docx [file BRB3-16-e71544-s002.docx]

Supplementary data: Codebook

| **Theme** | **Subthemes** | **Codes**  Codes and in vivo codes | **Definition** | **Example of quotes** |
| --- | --- | --- | --- | --- |
| Motivations | Hope | Previous experience with psychotherapy | This code describes positive experiences with psychotherapy that provided some participants with hope that the psychotherapy component within the trial would help them as well. | 1. Everything has something, I mean, it is good. It is always better for me to be in therapy than not. I have to say that. Simply because talking to someone about it is helpful. 939622 |
|  |  | Previous experience with psychedelic substances | This code describes positive experience with psychedelic experiences that provided some participants with hope that psychedelic experiences within the trial would be helpful to them as well. | 1. With the psilocybin, I had taken it in very small doses and therefore I was not on any kind of trip or something like that. But I’ve noticed that I was more mindful of my experience and my view. I also could enjoy life more, I was less in my head. It is difficult to explain, but in any case, it was a good feeling and it was not like I was impaired by it or something like that. 245533 |
|  |  | Return of hope | This code was used if participants were motivated by the wish that hope would return to their lives. | 1. Let me put it this way, helplessness and hopelessness are definitely clear symptoms that I also have. That is also very dominant. (...) The question of hope is somehow a bit difficult. I would very much like, well for me it would be a great success, to experience a reference experience for joy as part of this. That would be great, for example. 728931 |
|  |  | Medicalization | This code was used when the medical setting with safety aspects as well as what some participants perceived as “natural substances’” provided hope. | 1. Yes, I am definitely very excited, very confident, very optimistic, so I also think it's good that something like this exists in a medical setting, yes, and I'm looking forward to it and that gives me respect because I know that they are strong substances and I simply hope to be accompanied in the study and to always have a contact person if something goes wrong somehow. 245533 |
|  |  | Hope for relief | This code describes the hope for relief that some participants expressed. | 1. It seems to me like, well, from what I've read, it just sounds, it's given me hope. (....) And somehow that it's also a different kind of treatment, that it's not a drug that just kind of brings you down a bit or makes you a bit duller or something. 697803 |
|  |  | Study participation as “last straw” | This code describes the expressed motivation by many participants that the trial was hope for a last chance at symptom reduction. | 1. That is the last straw, a possibility, another form of therapy that I can access. 915567 |
|  | Demoralization | Treatment resistance | This code describes participants’ experiences of treatment failure with both psychotherapy and medication. | 1. The SSRIs have made it worse. No medication has worked, basically they just had side effects and I had terrible episodes when I discontinued them. Mhm. Well, really a complete failure. The complete therapy from 2007 until now has been a complete failure. 101668 |
|  |  | Chronic illness trajectory | This code describes the experience of chronic illness. | 1. The older you get, perhaps also when you somehow have less hope that things will get really good again at some point. 700135 |
|  |  | Hopelessness | This code describes the experience of hopelessness by participants. | 1. Yes, because otherwise I don't know what else I can do. I've already been to a day clinic, I've taken medication in maximum doses and well, I think there are only stronger antidepressants that I could take, but otherwise, yes, it just somehow feels... well, a bit hopeless. 245533 |
|  | Psychedelic experiences | Novelty of the experience | The code was used when the novelty and openness of the previous psychedelic experience was given as a motivating reason for wanting to participate in the trial. | 1. That was more like a hope for a bit of improvement and at the same time just seeing what comes. Because this experience was also completely new to me. 433013 |
|  |  | Ego death | This code describes motivation for the experience of Ego death through 5-MeO-DMT. | 1. What I missed in the psilocybin study was this dissolution of the ego and this switching off of the Default Mode Network, this 'I-am-not-there'. And that is something that scares me, but also interests me. 362090 |
|  |  | Curiosity | This code described the curiosity for the psychedelic experience. | 1. And that's certainly how the impulse arose in me. And there, yes sort of, can I say curiosity or something, yes perhaps to have a curiosity regarding whether there could be something that helps. 500212 |
|  |  | Previous participation in a psychedelic trial | This code described the experience by a few participants who had already participated in clinical trials using other psychedelic substances and were looking for an experience with a different substance. | 1. I participated in the psilocybin study, and for me, it didn't have a particularly good effect. I then took SSRIs again and saw again that it doesn’t help and even makes things worse for me. And (....) Yes, that is, I think, in the integration group of the psilocybin study... well, not during the group, but afterwards we talked and said: it is despair. You don't know where to go, you don't know where to turn. What I’ve tried so far hasn’t worked. (...) With psilocybin, it did do something, but it also made a lot of things worse for me right now, because I couldn't work through it further. 362090 |
|  | Social motivation | Recommendation from family and friends | This code was used if there was an active recommendation, not merely a supportive stance towards trial participation. | 1. I talked to a friend about it, and he's a psychotherapist himself, and he's had his own experience with the medication. 340023 |
|  |  | Support from family and friends | This code was used to describe a supportive stance towards the trial participation by the family and friends. | 1. I told my friends about it, my girlfriend too. They are open to it, it is not like… well some have made experience with substances as well. 655459 |
|  |  | Scepticism and stigma | This code describes a sceptical or stigmatized stance towards psychedelic substances by the social network. | 1. They think I am crazy that I want to participate. 245533 |
| Expectations | Symptom reduction | Better quality of life | This code describes participants expectations for better quality of life. | 1. Well actually a better quality of life again, at least what I had in average phases. 340023 |
|  |  | Management of expectations | This code describes management of expectations, especially regarding treatment effect, by lowering expectations. | 1. Um, let me put it this way: I have no expectations because I don't want my expectations to be disappointed. 362090 |
|  |  | Cure | This code describes participants’ expectation to be cured of symptoms after participating in a psychedelic trial. This code was used when the absence of symptoms was given. | 1. With psychedelics, there is indeed the expectation that after a limited number of single doses... a significant improvement occurs, perhaps a cure for some patients. 101668 |
|  |  | „Small changes“ | This code describes participants expectations for small changes in their symptoms. | 1. I don't know, maybe that this time it helps or at least brings a small improvement. That would be better than nothing. 790478 |
|  |  | Fundamental change | This code describes participants expectations for a fundamental change to their person; however, this code does not necessarily also entail cure or absence of symptoms. | 1. Well, first and foremost, of course, the desire for growth or improvement. I mean, I've had a few failed attempts, if you will. Of course, I also have a very long history of this depression, spanning decades, and for almost a year now, I've been in a relatively severe episode, sometimes worse than I've known before, and I thought, okay, if it's possible, it would be good if something fundamentally changed. And not, so to speak, maybe just a temporary relief, then see you next time or something. 942269 |
|  |  | Breakthrough | This code describes participants expectations for a breakthrough. This code was used to describe the rapid shift in comparison to fundamental change. | 1. At best, a breakthrough. 697803 |
|  | Expectation for mechanism of change | Wish for ego death | This code describes participants expectations the specific desire to experience the dissolution of the self/ego as a mechanism for healing. | 1. What I missed in the psilocybin study was this dissolution of the ego and this deactivation of the default mode network, this “I'm not there”. And that's something that scares me, but also interests me. 362090 |
|  |  | Connectedness with self and others | This code describes participants expectations for an experience that will restore a sense of connection to one's own self or the outside world/nature. | 1. In a way that I find access to myself again in a different way. More again, yes. 697803 2. Simply a feeling of being held in the world, to feel connected to the world. 700135 |
|  |  | Trust | This code describes participants expectations to regain a sense of trust or general confidence in life and the future. | 1. So for example there was the theme of trust, a kind of primal trust that things basically turn out positively or can turn out positively. That is something I haven't known for a very long time. I have a very negative view of many things. I often expect negative things quickly. (...) That was a theme that was one of the very present themes and that also helped me the months after, (.) to reconnect a bit with this trust in everyday life. 433013 |
|  |  | Discovering trauma | This code describes one participant’s expectation to discover childhood trauma. | 1. I don't have the feeling that I suffer from PTSD or something similar after [traumatic event], but perhaps I am repressing something. My [relative] revealed to me half a year ago that we were likely sexually abused by [person], which I also do not recall. Something I feared a bit in the beginning, when I first heard of the study, was that traumas could be broken open. Ultimately, it could actually be something positive if it uncovers something that perhaps should be therapeutically treated. 201105 |
|  |  | Emotional experience  and “working with experience” | This code describes participants expectations for the psychedelic experience to be emotional and to use this experience for further emotional work within therapy or outside of therapy. | 1. So, I really think it's very important, and I hope so too, that I somehow have the space or the opportunity and perhaps the support to work with it and explore it. 700135 |
|  |  | Change one’s view | This code describes participants expectations that the substance will alter the participants perspective on life. | 1. I hope that it changes my view of life or also certain things. That one doesn't ruminate so much. That something simply changes in the state of consciousness or in the ego-state, which could result in the depression becoming less. 7690478 |
|  |  | Generating hope through perspective change | This code describes participants expectations that the experience would instil hope by changing the perspective, allowing a different view. This code was used when the participant primarily wanted to gain hope. | 1. Well, at least a door opener, I would say. Because it is initially only a study participation, I don't see the perspective now that it becomes a permanent treatment or so, but simply just the opening of a perspective. And with that perhaps also a return of the feeling of hope or something. 500212 |
|  |  | Neurobiological change | This code describes participants expectations for the brain to change on some level (neuronal, functional, structural). | 1. And then, of course, I also found the effects that were described quite interesting, like the improved connectivity in the brain, for example. That's also something I wanted to try again, so to speak. 101668 |
|  | Therapists and setting | “No real psychotherapy” | This code describes participants expectations for the psychotherapy component of the trial to be less intense than psychotherapy. | 1. I have simply done so many therapies already that I don't know now whether the type of support, how it looks there now, it's probably not bad, but it probably doesn't go as deep as the therapies I've had now, so I expect perhaps less from that, I have to say honestly. 939622 |
|  |  | Documentation | This code describes participants expectations for witnessing and documentation of their experience within the trial. | 1. If something comes up, that it is at least documented in such a way that I can then process it subsequently in therapy. 201105 |
|  |  | Support and trust | This code describes participants expectations for expected support if they had challenging experiences. | 1. I expect them to support me in navigating what can be anxiety-provoking episodes. Above all, I need to be able to trust them, even though the treatment is still in an experimental stage and not yet fully proven. 340023 |
|  |  | Psychedelic experience: nice but not necessary | This code describes participants expectations for a psychedelic experienced therapist. | 1. Maybe that... I would almost say that it would be good if they had some personal contact with the substance. Well, not necessarily with the substance, but in that direction, so that... Otherwise, for example, I'd probably think that I'm talking to someone who has no idea what I'm talking about, you know what I mean? The thing that's so intense about psychedelics is that no matter how much you read about it or look at trip reports, you just can't imagine it until you've experienced it yourself, I'd say. 655459 |
|  |  | Empathy, containing, holding space and openness | This code describes participants expectations for therapy metaphors for empathetic, containing therapists, who would hold space open to experience. | 1. I hope that I feel safe and held in that environment. (...) Otherwise, I'd probably just do it alone. But I wouldn't dare to do that. I'm hoping that they'll provide a good setting. So that, within that framework unlike if I were to do it alone, I'll have the opportunity to, you know, actually work with it. I mean, through conversations. 433013 |
|  |  | Safety | This code describes participants expectations for safety provided by the therapists that was both in turn emotional and physical safety. | 1. I've had, I'd say, quite a few therapists… um… and I think I just expect to be given some kind of sense of security. You know, some people try to take matters into their own hands, they start taking things without talking to doctors or anything. For me, being part of a study or having doctors involved just makes me feel safe. It's about being given that sense of security, that feeling of control, and… yeah, and just a general sense of openness. 334224 |
|  |  | Professionalism | This code describes participants expectations for therapists to be professional. | 1. I would wish for this guidance to be as professional as possible. Excuse the openness, I don't expect very much else. I have simply had very different experiences over the decades, also made very good experiences, but also catastrophically bad experiences. 942269 |
|  | Expectations in retrospect | Disavowed expectations | This code describes one participant’s experience who disavowed their own expectations, saying that they had none, but actually expected symptom reduction. This code was used when expectations were not only consciously managed but when they were surprised that they were disappointed after the trial. | 1. Yes, well, I was disappointed because I think I had high hopes that it could make a big difference. But it was also on two levels. I was just generally curious, especially about psychedelics. I was interested in them anyway. Yes, and the fact that I did not benefit was, of course, disappointing. I think more than I expected. 700135 (post-trial) 2. Yes, but it didn't harm me. Luckily not, well, apart from the disappointment, which was of course intense, and I think it can be really bad if you have very... severe depression, and then... Yes, you've spent weeks preparing for it, and it's impossible not to have expectations. 700135 (post-trial) |
|  |  | Shifting expectations | This code describes participants expectations for a certain experience to work with, while later stating that they expected to just wanted to have tried every option available to them. | 1. But in the best case, another experience that I can build on later. 433013 (pre-trial) 2. Any form of improvement, however small, or if nothing improves, even just knowing that I have tried everything I can think of or know about. 433013 (post-trial) |
